# 368. Performance Characteristics of Sequencing Assays for Identification of the *SARS-CoV-2* Viral Genome

**DOI:** 10.1093/ofid/ofab466.569

**Published:** 2021-12-04

**Authors:** Danny Antaki, Mara Couto-Rodriguez, Tong Liu, Kristin Butcher, Esteban Toro, Bryan Höglund, Xavier O Jirau Serrano, Joseph Barrows, Bradley A Connor, Christopher Mason, Niamh B O’Hara, Dorottya Nagy-Szakal

**Affiliations:** 1 Twist Bioscience, South San Francisco, California; 2 Biotia, New York, New York; 3 Weill Cornell Medicine, New York, New York

## Abstract

**Background:**

As the SARS-CoV-2 (SCV-2) virus evolves, diagnostics and vaccines against novel strains rely on viral genome sequencing. Researchers have gravitated towards the cost-effective and highly sensitive amplicon-based (e.g. ARTIC) and hybrid capture sequencing (e.g. SARS-CoV-2 NGS Assay) to selectively target the SCV-2 genome. We provide an *in silico* model to compare these 2 technologies and present data on the high scalability of the Research Use Only (RUO) workflow of the SARS-CoV-2 NGS Assay.

**Methods:**

*In silico* work included alignments of 383,656 high-quality genome sequences belonging to variant of concern (VOC) or variant of interest (VOI) isolates (GISAID). We profiled mismatches and sequencing dropouts using the ARTIC V3 primers, SARS-CoV-2 NGS Assay probes (Twist Bioscience) and 11 synthesized viral sequences containing mutations and compared the performance of these assays using clinical samples. Further, the miniaturized hybrid capture workflow was optimized and evaluated to support high-throughput (384-plex). The sequencing data was processed by COVID-DX software.

**Results:**

We detected 101,432 viruses (27%) with > = 1 mismatch in the last 6 base pairs of the 3’ end of ARTIC primers; of these, 413 had > = 2 mismatches in one primer. In contrast, only 38 viruses (0.01%) had enough mutations ( > = 10) in a hybrid capture probe to have a similar effect on coverage. We observed that mutations in ARTIC primers led to complete dropout of the amplicon for 4/11 isolates and diminished coverage in additional 4. Twist probes showed uniform coverage throughout with little to no dropouts. Both assays detected a wide range of variants (~99.9% coverage at 5X depth) in clinical samples (CT value < 30) collected in NY (Spring 2020-Spring 2021). The distribution of the number of reads and on target rates were more uniform among specimens within amplicon-based sequencing. However, uneven genome coverage and primer dropouts, some in the spike protein, were observed on VOC/VOI and other isolates highlighting limitations of an amplicon-based approach.

**Conclusion:**

The RUO workflow of the SARS-CoV-2 NGS Assay is a comprehensive and scalable sequencing tool for variant profiling, yields more consistent coverage and smaller dropout rate compared to ARTIC (0.05% vs. 7.7%).

**Disclosures:**

**Danny Antaki, PhD**, **Twist Bioscience** (Employee, Shareholder) **Mara Couto-Rodriguez, MS**, **Biotia** (Employee) **Kristin Butcher, MS**, **Twist Bioscience** (Employee, Shareholder) **Esteban Toro, PhD**, **Twist Bioscience** (Employee) **Bryan Höglund, BS**, **Twist Bioscience** (Employee, Shareholder) **Xavier O. Jirau Serrano, B.S.**, **Biotia** (Employee) **Joseph Barrows, MS**, **Biotia** (Employee) **Christopher Mason, PhD**, **Biotia** (Board Member, Advisor or Review Panel member, Shareholder) **Niamh B. O’Hara, PhD**, **Biotia** (Board Member, Employee, Shareholder) **Dorottya Nagy-Szakal, MD PhD**, **Biotia Inc** (Employee, Shareholder)

